# Mesoporous Co*_x_*Sn_(1–*x*)_O_2_ as an efficient oxygen evolution catalyst support for SPE water electrolyzer

**DOI:** 10.1098/rsos.182223

**Published:** 2019-04-24

**Authors:** Gang Chen, Jiakun Li, Hong Lv, Sen Wang, Jian Zuo, Lihua Zhu

**Affiliations:** 1 College of Materials and Engineering, Hunan University, Changsha, Hunan 410082, People's Republic of China; 2 School of Automotive Studies, Tongji University, Shanghai 201804, People's Republic of China; 3 Clean Energy Automotive Engineering Center, Tongji University, Shanghai 201804, People's Republic of China

**Keywords:** Co*_x_*Sn_(1−*x*)_O_2_, IrO_2_, SPE water electrolyzer, oxygen evolution reaction

## Abstract

SPE water electrolysis is a promising method of hydrogen production owing to its multiple strengths, including its high efficiency, high product purity and excellent adaptability. However, the overpotential of the oxygen evolution reaction process and consumption of Ir during charging in SPE water electrolysis will inevitably result in large energy loss and then high cost. Under these circumstances, we propose a novel 40IrO_2_/Co*_x_*Sn_(1−*x*)_O_2_ (*x* = 0.1, 0.2, 0.3) anode catalyst, where the Co*_x_*Sn_(1−*x*)_O_2_ support is synthesized by a hydrothermal method and IrO_2_ is synthesized by a modified Adams fusion method. After modifying the component of Co*_x_*Sn_(1−*x*)_O_2_, the 40IrO_2_/Co*_x_*Sn_(1−*x*)_O_2_ exhibits an increased specific surface area, electrical conductivity and surface active sites. Moreover, a single cell is fabricated by Pt/C as cathode catalyst, 40IrO_2_/Co*_x_*Sn_(1−*x*)_O_2_ as anode catalyst and Nafion 117 membrane as electrolyte. The 40IrO_2_/Co_0.2_Sn_0.8_O_2_ exhibits the lowest overpotential (1.748 V at 1000 mA cm^−2^), and only 0.18 mV h^−1^ of voltage increased for 100 h durability test at 1000 mA cm^−2^. Consequently, Co*_x_*Sn_(1−*x*)_O_2_ is a promising anode electrocatalyst support for an SPE water electrolyzer.

## 1. Introduction

Hydrogen is regarded as one of the promising solutions for developing clean energy and solving the thorny environmental problems present on the Earth [1]. Water splitting for hydrogen generation is a major component of modern clean energy technologies [2], such as water-alkali electrolyzers [3], solid polymer electrolyte (SPE) water electrolyzers [4] and photocatalytic/photo-electrochemical water splitting [5–8]. SPE water electrolyzers offer us an effective and simple method to produce hydrogen through reusing the surplus electric power generated by renewable energy (such as wind and photovoltaic power) [9]. As a result, SPE water electrolyzers have gained a lot of research attention [10–12]. Nevertheless, the conspicuous weakness of SPE water electrolysis should not be ignored, including the majority increases of the activation overpotential in the oxygen evolution reaction (OER) process [13]. Although some highly active and stable noble metal-based catalysts have been developed, such as RuO_2_ and IrO_2_ for OER, these materials are still far from large-scale application because of their high cost and scarcity. Therefore, multiple studies have been devoted to develop novel, highly efficient and low-cost catalysts (e.g. La_2_NiMnO_6_ [14], Ni-Fe-layered double hydroxide [15] and Ternary Ni–Co–Fe blue analogue [16]). However, these new catalysts too have drawbacks, such as a complicated preparation process and poor durability*.* Additionally, many research efforts have been made to reduce the amount of these noble metals, such as designing their bulk structural parameters (i.e. grain size, morphology, and dimensions) [17–19], tailoring composition (such as introducing foreign elements or oxides into the structure of noble metal catalysts) [20], and adopting supports (i.e. carbon-based [21], SnO_2_ [22], TiO_2_ [10], TiC [23]).

It is well known that an appropriate support will favour a noble metal-based material to achieve a better dispersion and greater surface area, which not only reduces the usage of noble metal but also maintains high activity for the catalysts [23]. As supports, carbon-based materials have recently received a lot of attention due to their high specific surface area and excellent electric conductivity. Unfortunately, carbon-based materials are easily electrochemically oxidized at potential above 0.206 V versus SHE [24], leading ultimately to the unsustainability of carbon-based supports in SPE water electrolyzer [25]. Therefore, the development of a highly stable and active support for the OER is still a research focus. Tin oxide (SnO_2_), as a corrosion-resistant support, has been reported to promote the dispersion of noble metal-based materials and provide more surface active sites of catalysts [26]. Wang *et al*. reported that SnO_2_ in the IrO_2_/SnO_2_ catalyst could act as a Brønsted base and accept protons from the IrO_2_ sites, which is favourable to the enhancement of catalytic performance of IrO_2_/SnO_2_ catalyst [22]. Unfortunately, the current reported OER activity of SnO_2_-based supports is still not significant because of the poor electronic conductivity of SnO_2_. Heteroatom doping is a method of enhancing the catalytic activities of catalysts due to the charge delocalization mechanism [27]. Co is a transition element with incomplete electron shell and possesses interesting catalytic properties [28–30]. As far as we know, the effect on the electrocatalytic activity of SnO_2_ with varying Co content doped as support for IrO_2_ has been rarely studied.

In this study, IrO_2_ supported by mesoporous Co*_x_*Sn_(1−*x*)_O_2_ (*x* = 0.1, 0.2, 0.3) as an anode catalyst for SPE water electrolysis has been exploited. Co*_x_*Sn_(1−*x*)_O_2_ (*x* = 0.1, 0.2, 0.3) is successfully synthesized by a hydrothermal method and IrO_2_ is synthesized by using a modified Adams fusion method [4,31]. The structure, morphology, specific surface area, electrical conductivity and surface active sites of the prepared samples have been thoroughly determined by various characterization techniques. The electrocatalytic activity of the prepared samples as anode catalysts in single cells is also tested. The prepared samples with Co doping show low overpotential (1.748 V at 1000 mA cm^−2^) and excellent stability.

## 2. Experimental section

### 2.1. Preparation of Co-doped SnO_2_ support

The supports of Co*_x_*Sn_(1−*x*)_O_2_ (*x* = 0, 0.1, 0.2, 0.3) were synthesized by the hydrothermal method and then processed by heat treatment. One gram of hexadecyl trimethylammonium bromide (Sinopharm, Shanghai, China) was dissolved in a mixture of 20 ml of ethanol (Sinopharm) and 20 ml distilled water. Then, significant amounts of tin chloride (Sinopharm) and cobalt acetate (Sinopharm) (molar ratio (Sn^2+^ + Co^2+^): CTAB = 1 : 1.05) were added to the above solution, and continuously stirred to get a homogeneous solution. A certain amount of ammonia water (Sinopharm) was added dropwise to the solution under vigorous stirring at room temperature. The Ph value of the solution was maintained at 9 and under stirring for 2 h. After it was stirred, the mixture was transferred to a stainless Teflon-lined 100 ml autoclave and kept at 180°C for 24 h in an oven. The resulting yellow precipitate was collected by centrifugation, washed with distilled water and ethanol several times to remove the impurities, and then dried in a vacuum oven at 60°C. The dried samples were processed under 350°C for 5 h in a muffle furnace. After cooling to room temperature, Co*_x_*Sn_(1−*x*)_O_2_ nanoparticles were finally obtained.

### 2.2. Preparation of supported catalysts

In this work, the IrO_2_ loading was applied as 40 wt %, 0.196 g chloroiridic acid (Hesen, Shanghai, China), 5 g of ultrafine sodium nitrate (Sinopharm) and 0.12 g as-prepared support powder were added into 10 ml of isopropyl alcohol (Sinopharm), which was stirred to obtain uniform suspension. Then the suspension was ground for 6 h by planetary ball milling. The obtained slurry was dried in a vacuum oven at 60°C, and treated at 400°C for 1 h in a muffle furnace with a heating rate of 5°C min^−1^. After heat treatment, the powder was washed in turn with 0.1 M HCl, distilled water, and ethanol to eliminate residual impurities, and dried in a vacuum oven at 70°C overnight. The resulting material was denoted as 40IrO_2_/Co*_x_*Sn_1−*x*_O_2_ (*x* = 0.1, 0.2, 0.3). The preparatory method of unsupported IrO_2_ was similar to that of the 40IrO_2_/Co*_x_*Sn_1−*x*_O_2_ samples.

### 2.3. Physical characterization

X-ray diffraction (XRD) was performed to obtain the crystal structure and phase purity of all the prepared samples using a Bruker D8 Advance X-ray diffractometer (Bruker, Karlsruhe, Germany) with a Cu-Ka radiation source (*λ* = 0.154056 nm). Transmission electron microscopy (TEM) and high-resolution TEM (HR-TEM) images were carried on a JEOL 2010F microscope (JEOL, Tokyo, Japan). The specific surface area and pore size distribution of the as-prepared samples were recorded with the measurement of nitrogen adsorption isotherm at 77 K using a Micromeritics ASAP 2020 analyzer (Micromeritics, Norcross, Georgia, USA). Electrical conductivity measurements were carried out on cylindrical pellets compressed from the powder samples at 30 MPa between two copper electrodes. The substrate area was restricted to 1 cm^2^ while the thickness of the pellet was measured by a Vernier caliper. The value of resistivity was immediately measured by a JG-ST2258A resistivity tester (Jingge Electronic, Suzhou, Jiangsu, China) by inputting the thickness-area ratio as a parameter, followed by conversion to conductivity.

### 2.4. Electrochemical characterization

The half-cell electrochemical evaluation of different samples was investigated by a three-electrode measurement in the N_2_-saturated 0.5 M H_2_SO_4_ electrolyte. A reversible hydrogen electrode (RHE) and a platinum wire acted as the reference and counter electrode, respectively. The working electrode was prepared by a catalyst layer coating on the glassy carbon disc (GCE, 5.6 mm in diameter). Briefly, the catalyst layer was fabricated as follows: 12.46 mg of 40IrO_2_/Co*_x_*Sn_1−*x*_O_2_ powder was dispersed in 2 ml methanol/Nafion (50 : 1, wt.%) mixed solution and uniform ultrasound solution was obtained by ultrasound. The loadings of all samples on glassy carbon were controlled at 0.1 mg cm^−2^. Cyclic voltammetric (CV) measurements were performed on a CHI 760E Electrochemical Workstation at a scanning rate of 50 mV s^−1^ between 0.05 and 1.35 V_RHE_. The potential range of the linear sweeping voltammetry (LSV) curve was from 1.4 to 1.65 V versus RHE at a scan rate of 50 mV s^−1^, and the rotation rate of the working electrode was 1600 rpm.

**Scheme 1.**
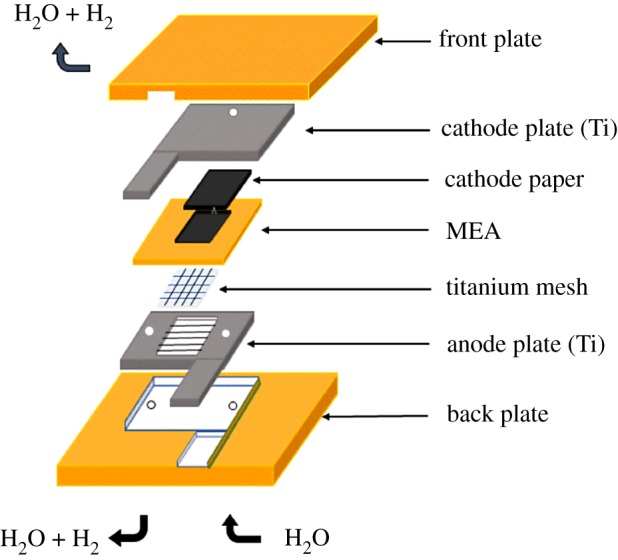
Schematic of the SPE water electrolyzer structure.

The membrane electrode assemblies (MEAs) were prepared by the spray method and assembled using a Nafion117 membrane (DuPont, Wilmington, Delaware, USA) adopted as an SPE, the prepared samples were used as anodic electrocatalysts and a commercial 60 wt.% Pt/C (Johnson Matthey, London, UK) catalyst acted as cathodic electrocatalyst. Prior to the assembly, the Nafion117 membrane was cleaned by H_2_O_2_ solution, distilled water, and H_2_SO_4_ solution at 80°C for 1 h for each step. The sprayed catalyst inks were fabricated by the mixture of the obtained samples, Nafion solution, isopropyl alcohol and deionized water, and sonicated for 2 h to get a homogeneous suspension. The Nafion loading on each side of the membrane was maintained at 25 wt.%. The noble metal Ir and Pt loading on the membrane was 2.5 mg cm^−2^ for the anode and 0.5 mg cm^−2^ for the cathode, respectively. Finally, the MEAs (with an effective area of 3.645 cm^2^) were assembled into a home-made single cell water electrolyzer, as shown in the schematic diagram in scheme 1. Ti mesh and plates were made as current collector and bipolar plate for the anode side, respectively. Carbon paper and Ti plates were used as current collector and bipolar plate for the cathode, respectively. Deionized water with a preheated 80°C temperature was pumped by a peristaltic pump to the anode compartment. At atmospheric pressure, the polarization curves of the single cells were measured by a Motech LPS305 programmatic DC power supply. The electrochemical impedance spectrums (EIS) for the single cells were conducted at 0.3 A cm^2^ in the frequency range of 0.1 Hz to 10 kHz (amplitude = 80 mV) and recorded with a Solartron Analytical 1260 impedance analyzer coupled to a Solartron Analytical 1287 potentiostat.

## 3. Results and discussion

### 3.1. Physical characterization

**Figure 1.**
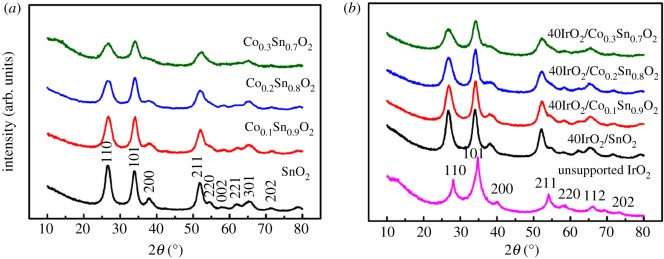
XRD patterns of the (*a*) Co*_x_*Sn_1−*x*_O_2_; (*b*) 40IrO_2_/Co*_x_*Sn_1−*x*_O_2_ (*x* = 0, 0.1, 0.2, 0.3) and unsupported IrO_2_.

The XRD patterns of the prepared samples are shown in figure 1. Figure 1*a* shows that all the diffraction peaks of SnO_2_ are matched well with the characteristic peaks of tetragonal rutile structure (JCPDS 41–1445) [32]. The diffraction peaks located at approximately 26.6°, 33.9°, 37.9°, 51.8°, 54.7°, 57.8°,62.6°, 65.9°, 71.3° and 78.7° represent the (110), (101), (200), (211), (220), (002), (221), (301), (202) and (321) planes, respectively. It is noted that the diffraction peaks of SnO_2_ doped with varying Co content show similar shapes to the pure SnO_2_, and no second phase about Co is detected, which confirms that Co ions were successfully doped into SnO_2_ [33]. Figure 1*b* displays the XRD patterns of the unsupported IrO_2_ and 40IrO_2_/Co*_x_*Sn_1−*x*_O_2_ (*x* = 0, 0.1, 0.2, 0.3). The typical peaks of the unsupported IrO_2_ could be matched well with the tetragonal rutile structure [31]. After IrO_2_ supported on Co*_x_*Sn_1−*x*_O_2_, the 40IrO_2_/Co*_x_*Sn_1−*x*_O_2_ exhibit similar shapes with Co*_x_*Sn_1−*x*_O_2_, implying that the loaded IrO_2_ will not affect the crystal structure of Co*_x_*Sn_1−*x*_O_2_ by our presented modified Adams fusion treatment. The lattice parameters (a = b, c) of SnO_2_ and Co*_x_*Sn_1−*x*_O_2_ (as listed in table 1) decrease with the increasing Co-doping concentration. This is because the radius of Co^2+^ (0.072 nm) is smaller than that of Sn^4+^ (0.083 nm) at a coordination number of 6 [34]. Furthermore, the grain sizes of all the obtained samples were calculated using the by Debye­–Scherrer equation, *L* = K*λ*/(*β*cos*θ*), where K is the constant (0.89), *λ* is the wavelength of the X-ray radiation (Cu Kα = 0.15406 nm), *β* is the line width at half maximum height and *θ* is the diffracting angle. The calculated grain sizes of all the prepared samples are listed in table 1. The grain sizes of Co*_x_*Sn_1−*x*_O_2_ (*x* = 0, 0.1, 0.2, 0.3) gradually decrease from 8.28 nm to 5.52 nm with the Co content increasing, which may be attributed to the fact that the highly Co doped induced a segregation at the grain boundaries, which leads to a decrease in the grain size [35].

**Table 1.** The lattice constant, grain sizes, BET surface area and BJH adsorption average pore diameter results of Co*_x_*Sn_1−*x*_O_2_ (*x* = 0, 0.1, 0.2, 0.3).

	lattice constant				
samples	a = b (Å)	c (Å)	grain size (D) nm	BET surface area (m^2^ g^−1^)	BJH adsorption average pore diameter (nm)
SnO_2_	4.7367	3.1908	8.28	72.19	7.88
Co_0.1_Sn_0.9_O_2_	4.7194	3.1849	7.08	104.01	8.48
Co_0.2_Sn_0.8_O_2_	4.7084	3.14430	6.54	131.92	10.59
Co_0.3_Sn_0.7_O_2_	4.6918	3.1273	5.52	132.22	7.47

**Figure 2.**
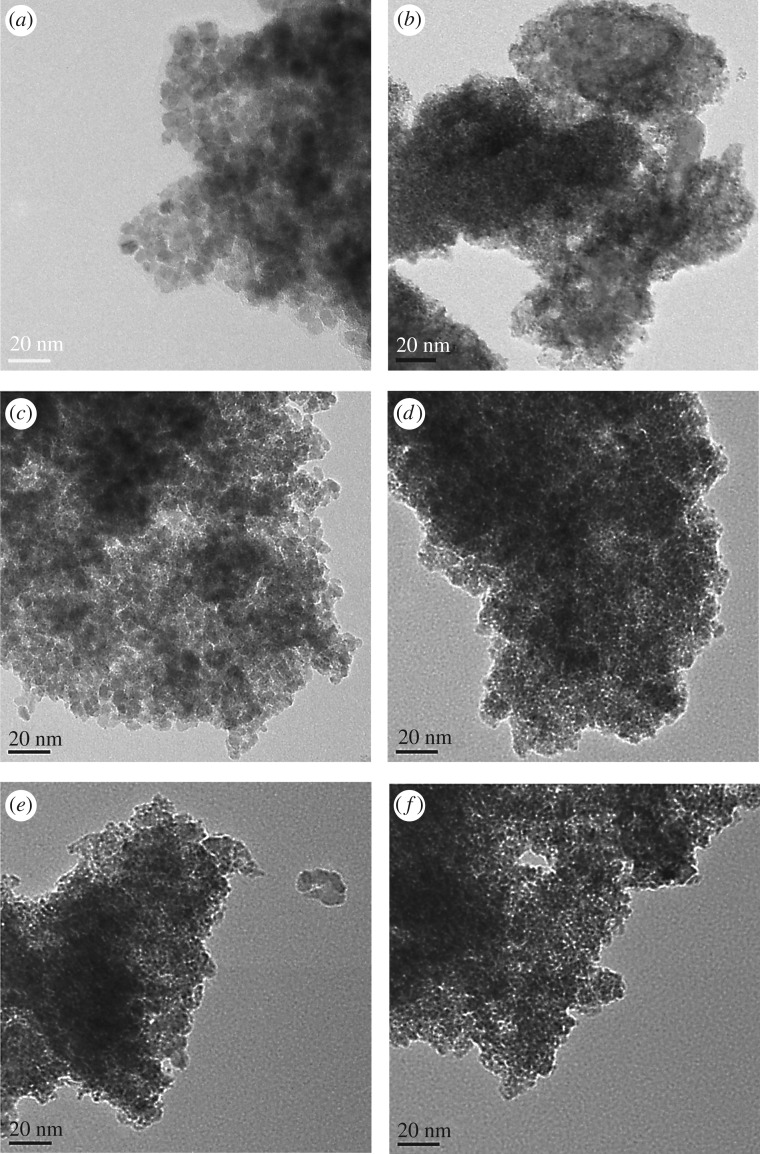
TEM images of (*a*) unsupported IrO_2_, (*b*) SnO_2_, (*c*) 40IrO_2_/SnO_2_ (*d*) 40IrO_2_/Co_0.1_Sn_0.9_O_2_, (*e*) 40IrO_2_/Co_0.2_Sn_0.8_O_2_ and (*f*) 40IrO_2_/Co_0.3_Sn_0.7_O_2_.

**Figure 3.**
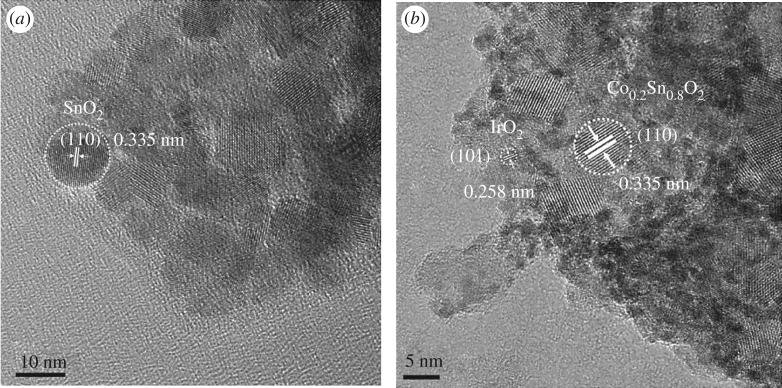
HR-TEM images of (*a*) SnO_2_ and (*b*) 40IrO_2_/Co_0.2_Sn_0.8_O_2_.

The morphologies and particle sizes of the 40IrO_2_/Co*_x_*Sn_1−*x*_O_2_ (*x* = 0, 0.1, 0.2, 0.3) and unsupported IrO_2_ were characterized by the TEM images. As shown in figure 2*a*, the prepared SnO_2_ is in an irregular shape and the average particle size is approximately 10.2 nm. Figure 2*b* shows that the unsupported IrO_2_ exhibits a quasi-spherical shape and serious aggregation of nanoparticles with a broad particle size distribution, which would result in a poor catalytic activity. In contrast to the unsupported IrO_2_, the IrO_2_ nanoparticles supported on Co*_x_*Sn_1−*x*_O_2_ present a quasi-spherical shape with sub 3 nm sizes as the darker dots in figure 2*c*–*f*. Meanwhile, IrO_2_ nanoparticles are observed to be well-dispersed on the Co*_x_*Sn_1−*x*_O_2_ supports. It is noted that the particle sizes of Co*_x_*Sn_1−*x*_O_2_ supports decrease gradually with the increase of the Co-doped content, which is in agreement with the XRD results and previous report [34]. The reduced Co*_x_*Sn_1−*x*_O_2_ particle sizes would expose more surface area for the dispersion of IrO_2_ and avoid the serious aggregation of IrO_2_ nanoparticles. Therefore, the catalytic activities of prepared samples could be anticipated to enhance with the Co-doped content. Figure 3 exhibits the HR-TEM images of SnO_2_ and 40IrO_2_/Co_0.2_Sn_0.8_O_2_. The HR-TEM image in (figure 3*a*) confirms the SnO_2_ particles present an irregular shape with a mean particle size of about 10.2 nm. The lattice fringe is about 0.335 nm, corresponding to the (110) planes of SnO_2_. The HR-TEM image of figure 3*b* reveals the IrO_2_ in darker dots with the mean particle size of about 2.25 nm, and it disperses evenly on the surface of Co_0.2_Sn_0.8_O_2_. The lattice fringes are about 0.258 and 0.335 nm corresponding to the (101) plane of IrO_2_ and (110) plane of Co_0.2_Sn_0.8_O_2_, respectively. Additionally, the presence, relative amount and homogeneous distribution of the Ir and Co elements in these samples were verified by EDX mapping (electronic supplementary material, figure S1–S5). According to the obtained results, the atomic ratio of Co/Sn is around 8.1 for Co_0.1_Sn_0.9_O_2_, 4.0 for Co_0.2_Sn_0.8_O_2_ and 2.7 for Co_0.3_Sn_0.7_O_2_, which is consistent with the designed cobalt content.

**Figure 4.**
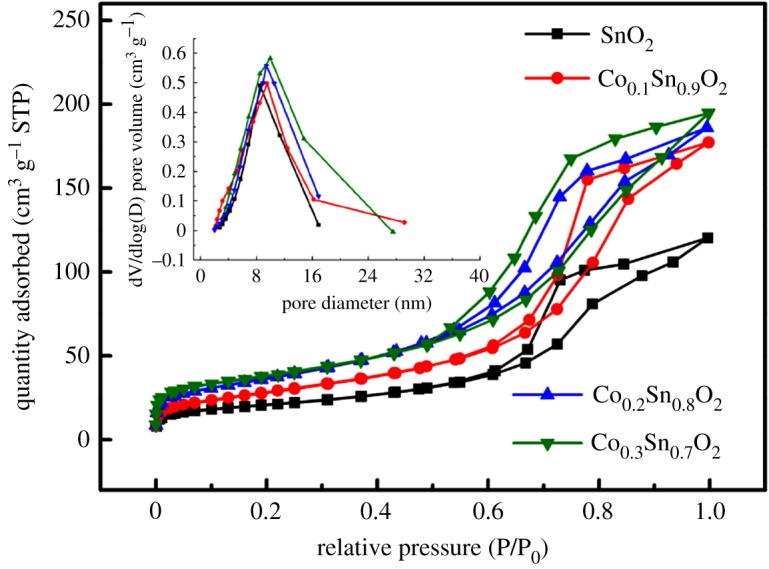
N_2_ adsorption isotherms and pore size distributions (inset) of the Co*_x_*Sn_1−*x*_O_2_ (*x* = 0, 0.1, 0.2, 0.3) supports.

It is well known that the specific surface areas play an important role in the catalytic activity of a catalyst. Thus, to identify the impact of Co doping and IrO_2_ loading on the specific surface areas and pore size of SnO_2_, nitrogen adsorption–desorption measurements were carried out. Figure 4 and electronic supplementary material, figure S7 represent the N_2_ adsorption–desorption isotherms of the Co*_x_*Sn_1−*x*_O_2_ and 40IrO_2_/Co*_x_*Sn_1−*x*_O_2_ (*x* = 0, 0.1, 0.2, 0.3), the inset are the corresponding Barrett–Joyner–Halenda (BJH) pore size distribution curves. Typical Langmuir type IV with an inherent hysteresis loop at relative high pressure is detected for all samples, which suggests that the pores between the nanoparticles are mainly constructed by mesoporous structures. The specific surface areas of the samples are calculated by Brunauer–Emmett–Teller (BET). It can be seen that the specific surface areas increase significantly from 72.19 m^2^ g^−1^ for SnO_2_ to 104.01, 131.92 and 139.22 m^2^ g^−1^ for Co_0.1_Sn_0.9_O_2_, Co_0.2_Sn_0.8_O_2_ and Co_0.3_Sn_0.7_O_2_, respectively (table 1). The enhancement of specific surface areas of Co*_x_*Sn_1−*x*_O_2_ might be attributed to the decreased particles sizes resulting from Co-doped SnO_2_, as demonstrated by the TEM results. As listed in electronic supplementary material, table S1, the specific surface areas for 40IrO_2_/SnO_2_, 40IrO_2_/Co_0.1_Sn_0.9_O_2_, 40IrO_2_/Co_0.2_Sn_0.8_O_2_ and 40IrO_2_/Co_0.3_Sn_0.7_O_2_ are 53.21, 87.54, 91.25, 93.06 m^2^ g^−1^, respectively. The specific surface areas of Co*_x_*Sn_1−*x*_O_2_ are higher than those of 40 IrO_2_/Co*_x_*Sn_1−*x*_O_2_ (*x* = 0.1, 0.2, 0.3), which might be due to the pore blocking of Co*_x_*Sn_1−*x*_O_2_ samples with IrO_2_ loading. From pore size distributions curves, the pore sizes of Co*_x_*Sn_1−*x*_O_2_ (*x* = 0, 0.1, 0.2, 0.3) samples are mainly about 9–10 nm, whereas the mainly pore sizes of 40IrO_2_/Co*_x_*Sn_1−*x*_O_2_ (*x* = 0, 0.1, 0.2, 0.3) samples are mainly about 3–5 nm and 6–7 nm, which could be a result of the IrO_2_ nanoparticles that occupied a certain amount of pore volume, resulting in a decrease in the pore size [36]. The Co-doped SnO_2_ samples exhibit a high specific surface area and evident porosity, which might be advantageous for the efficient catalytic performance of the prepared samples.

**Figure 5.**
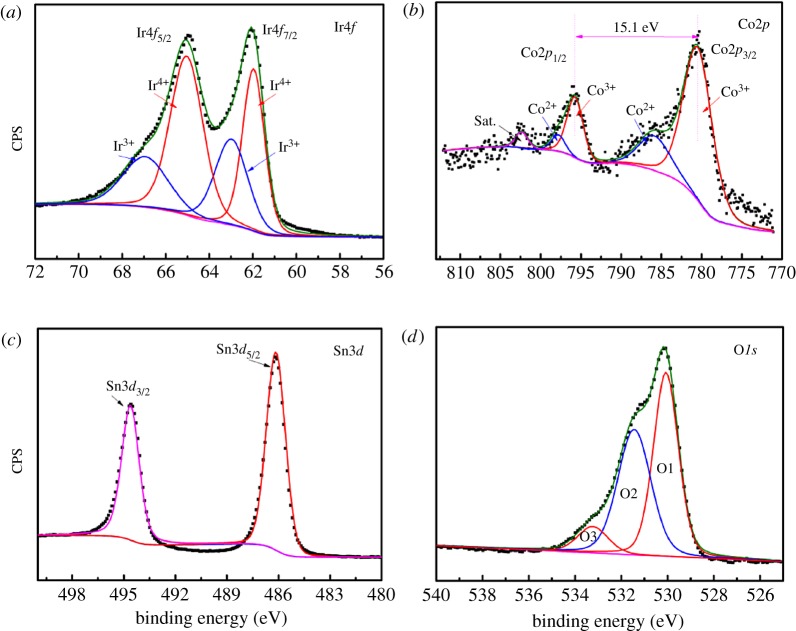
The high-resolution XPS spectrum of (*a*) Ir*4f*, (*b*) Co*2p*, (*c*) Sn*3d* and (*d*) O*1s* in 40IrO_2_/Co_0.2_Sn_0.8_O_2_.

The XPS spectra were evaluated to reveal the elemental states of each element in the 40IrO_2_/Co_0.2_Sn_0.8_O_2_ sample, as shown in figure 5. The high-resolution Ir4*f* spectrum (figure 5*a*) shows spin–orbit doublets at approximately 61.9 eV and 64.9 eV which can be classified as Ir4*f*_7/2_ and Ir4*f*_5/2_, respectively [37]. The binding energy of Ir4*f*_7/2_ at 61.9 and Ir4*f*_5/2_ at 64.9 eV is in the form of the Ir^4+^ and Ir4*f*_7/2_ at 62.9 and Ir4*f*_5/2_ at 65.9 eV is in accordance with Ir^3+^. Clearly, the atomic ratio of Ir^4+^ is higher than that of Ir^3+^, the ratio of Ir^4+^/Ir^3+^ is 1.42. This indicates that the majority of Ir element in the crystal lattice is Ir^4+^ cations. The Co2*p* spectrum (figure 5*b*) is deconvoluted into two main components at 781.2 eV for Co2*p*_3/2_, 797.8 eV for Co2*p*_1/2_, and two satellite peaks (noted as ‘Sat.’) are also detected. The presented satellite peaks and the difference of 15.1 eV of the Co2*p*_3/2_ and Co2*p*_1/2_ imply that the majority of cobalt is in the states of Co^2+^.[38] figure 5*c* exhibits the high resolution of Sn3*d* spectrum. The peaks located at about 487.3 eV and 495.8 eV are the representatives of Sn3*d*_5/2_ and Sn3*d*_3/2_, and no other peak is detected, confirming the chemical state of Sn is only in tetravalence [39]. The O 1 s spectrum (figure 4*d*) could be divided into three major peaks: O1 (approx. 529.4 eV), O2 (approx. 531.5 eV) and O3 (approx. 532.9 eV), which are according to the metal-oxygen bonding, oxygen vacancies and hydroxyl species of water molecules adsorbed on the surface, respectively [40].

High electrical conductivity of support is favourable to the supported catalysts for the enhancement of catalytic performance. Prior to the catalytic performance test, the electrical conductivities of all the prepared samples were measured and listed in electronic supplementary material, table S2. The electrical conductivities of SnO_2_, Co_0.1_Sn_0.9_O_2_, Co_0.2_Sn_0.8_O_2_ Co_0.3_Sn_0.9_O_2_, 40IrO_2_/SnO_2_, 40IrO_2_/Co_0.1_Sn_0.9_O_2_, 40IrO_2_/Co_0.2_Sn_0.8_O_2_, 40IrO_2_/Co_0.3_Sn_0.9_O_2_ and unsupported IrO_2_ are 1.95 × 10^−6^, 2.02 × 10^−5^, 9.51 × 10^−5^, 6.94 × 10^−5^, 6.08 ×10^−2^, 3.15 × 10^−1^, 1.02 × 10^0^, 8.13 × 10^−1^ and 1.02 × 10^1^ S cm^–1^, respectively. The electrical conductivities of Co-doped SnO_2_ are much higher than those of the pure SnO_2_, suggesting a favourable effect of the Co dopant on the improvement of the SnO_2_ conductivity. After the IrO_2_ loading, the conductivity of 40IrO_2_/Co*_x_*Sn_1−*x*_O_2_ (*x* = 0.1, 0.2, 0.3) is enhanced because of the excellent electrical conductivity of IrO_2_. However, the electrical conductivities of Co_0.3_Sn_0.9_O_2_ and 40IrO_2_/Co_0.3_Sn_0.9_O_2_ are lower than those of Co_0.2_Sn_0.8_O_2_ and 40IrO_2_/Co_0.2_Sn_0.8_O_2_, which might be due to the increasing impurity scattering centres with the enhancement of Co contents that impede the electron transport, decrease the carrier mobility and reduce electrical conductivity [41].

### 3.2. Electrochemical properties

**Figure 6.**
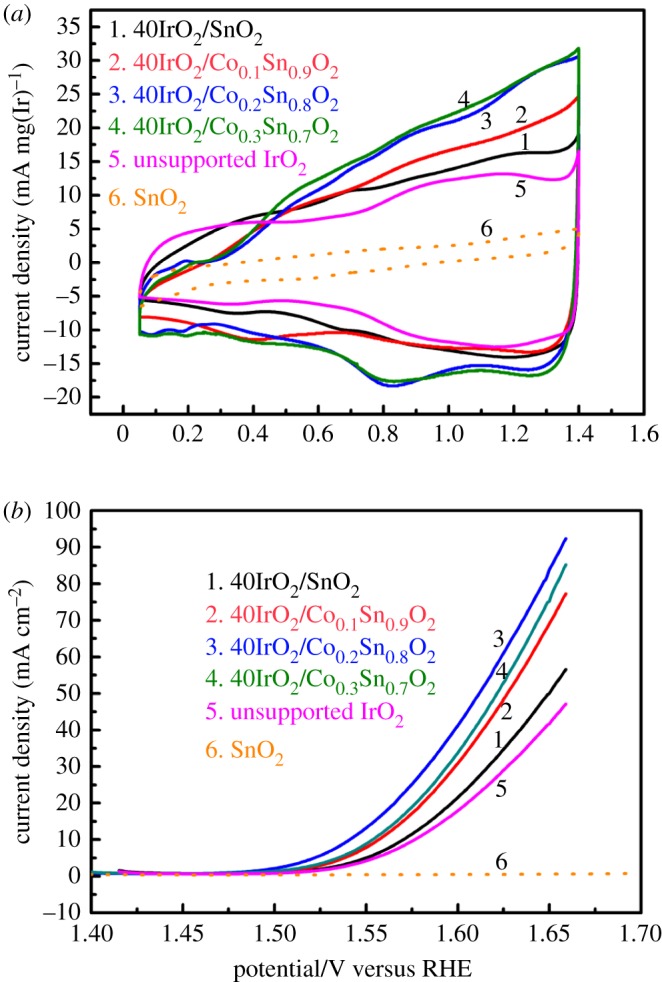
(*a*) Cyclic voltammetry curves of 40IrO_2_/Co*_x_*Sn_1−*x*_O_2_, pristine SnO_2_ and unsupported IrO_2_ in N_2_-saturated 0.5 M H_2_SO_4_ solution at a scan rate is 50 mV s^−1^ and (*b*) LSV curves of 40IrO_2_/Co*_x_*Sn_1−*x*_O_2_, pristine SnO_2_ and unsupported IrO_2_.

To evaluate the effect on the surface active sites of catalysts with Co doping, the cyclic voltammograms (CVs) of 40IrO_2_/Co*_x_*Sn_1−*x*_O_2_ were measured in N_2_-saturated 0.5 M H_2_SO_4_ at a scan rate of 50 mV s^−1^, as shown in figure 6*a*. For comparison, the CVs of the pristine SnO_2_ and unsupported IrO_2_ were also tested at the same condition, and the current densities of all the samples were normalized to the IrO_2_ loading. The pristine SnO_2_ shows very low current densities because of the poor electrocatalytic activity. The CVs of Co*_x_*Sn_1−*x*_O_2_ samples were also tested (electronic supplementary material, figure S6 (a)) and exhibited higher current densities with Co doping than that of SnO_2_, but still unsatisfactory. The shapes of the voltammogram of 40IrO_2_/Co*_x_*Sn_1−*x*_O_2_ are similar to those of the unsupported IrO_2_ but with a higher current density. The CVs of all samples present a broad redox peak of the reversible oxidation and reduction on the IrO_2_ surface, which suggests a typical pseudo-capacitive behaviour. The voltammetric charge of 40IrO_2_/Co*_x_*Sn_1−*x*_O_2_ and unsupported IrO_2_, as a function of scan rates, was calculated by the following [42]:

3.1 $$Q=\int_{E_{0}}^{E}\frac{\left|I\right|}{\nu m_{Ir}}\cdot dE,$$

where *I* is the current density obtained in CV curves, *v* = 50 mV s^−1^ is the scan rate, *m_Ir_* is the loading of noble metal Ir on the glassy carbon electrode, *E* is the scan potential between –0.148 and 1.15 versus Ag/AgCl. The sequence is 40IrO_2_/Co_0.3_Sn_0.7_O_2_ (333.2 C g(Ir)^−1^) > 40IrO_2_/Co_0.2_Sn_0.8_O_2_ (314.3 C g(Ir)^−1^) > 40IrO_2_/Co_0.1_Sn_0.9_O_2_ (252.6 C g(Ir)^−1^) > 40IrO_2_/SnO_2_(238.5 C g(Ir)^−1^) > IrO_2_ (203.5 C g(Ir)^−1^). This confirms the positive effect on the increment of surface active sites with Co doping. The enhancement of surface active sites could be ascribed to the decreased particle sizes of SnO_2_ supports that provide more sites for IrO_2_ dispersion. Consequently, according to the calculated voltammetric charge, it is expected that the OER catalytic activity of the prepared samples would be improved as the Co-doped samples. The overall OER performance of the samples will be discussed in the following single cell test results. Figure 6*b* shows the LSV polarization curves of the pristine SnO_2_, unsupported IrO_2_ and 40IrO_2_/Co*_x_*Sn_1−*x*_O_2_ samples in N_2_-saturated 0.5 M H_2_SO_4_ at a scan rate of 50 mV s^−1^. The potentials at the current density of 10 mA cm^−2^ are listed in table 2. The measured potentials are 1.577, 1.570, 1.557, 1.541 and 1.554 V versus RHE for unsupported IrO_2_, 40IrO_2_/SnO_2_, 40IrO_2_/Co_0.1_Sn_0.9_O_2_, 40IrO_2_/Co_0.2_Sn_0.8_O_2_ and 40IrO_2_/Co_0.3_Sn_0.7_O_2_, respectively. It is clearly observed that 40IrO_2_/Co_0.2_Sn_0.8_O_2_ reveals the lowest overpotential on mass activity, indicating Co-doped support could favour the increment of the active substance and enhance the OER performance. Electronic supplementary material, figure S6 (b) displays the LSV polarization curves of the Co*_x_*Sn_1−*x*_O_2_ samples. It is clearly observed that the overpotential of Co*_x_*Sn_1−*x*_O_2_ samples is higher than that of 40IrO_2_/Co*_x_*Sn_1−*x*_O_2_ samples, indicating the low catalytic activity of supports.

**Table 2.** The obtained values of voltammetric charge (C/g), the potentials at 10 mA cm^−2^, cell potential at 1 A cm^−2^, *R*_Ω_ and *R*_ct_ of the prepared samples.

		LSV		EIS (mΩ cm^2^)	
samples	voltammetric charge (C g^−1^)	The potentials at 10 mA cm^−2^	cell potential at 1 A cm^−2^	*R*_Ω_	*R*_ct_
40IrO_2_/SnO_2_	238.5	1.570	1.847	152.0	40.3
40IrO_2_/Co_0.1_Sn_0.9_O_2_	252.6	1.557	1.812	114.2	37.0
40IrO_2_/Co_0.2_Sn_0.8_O_2_	314.3	1.541	1.748	74.7	32.1
40IrO_2_/Co_0.3_Sn_0.7_O_2_	333.2	1.554	1.77	85.3	34.3
unsupported IrO_2_	203.5	1.577	1.713	63.1	52.6

### 3.3. Electrolysis cell performance

**Figure 7.**
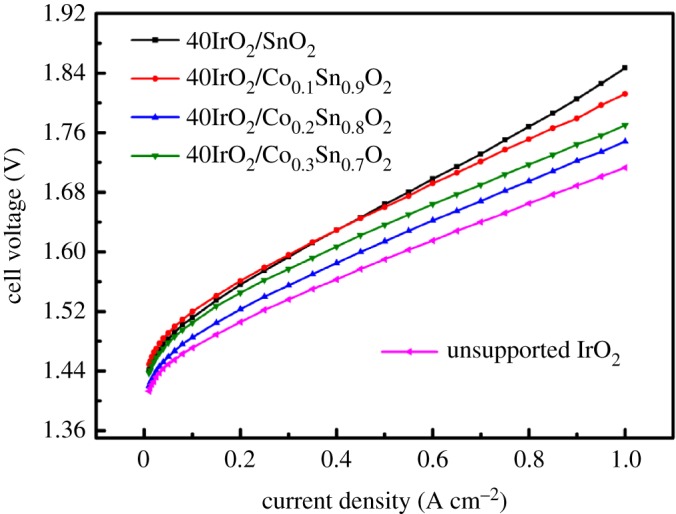
Polarization curves of single cells equipped with 40IrO_2_/Co*_x_*Sn_1−*x*_O_2_ (*x* = 0, 0.1, 0.2, 0.3) and unsupported IrO_2_ at 80°C.

After the MEAs assembled in single cells, the OER performance of the 40IrO_2_/Co*_x_*Sn_1−*x*_O_2_ catalysts was further characterized by I-V polarization measurement from 0.01 to 1 A cm^−2^ at 80°C, as displayed in figure 7. Again, the OER performance of unsupported IrO_2_ was detected at the same condition for comparison. For the low current density (less than 0.1 A cm^−2^), the cell voltage of the 40IrO_2_/Co*_x_*Sn_1−*x*_O_2_ increases and unsupported IrO_2_ increases nonlinearly along the current density, which is mainly affected by activation polarization. Once the polarization current density increases gradually, the disparity in the OER performance is mainly due to ohmic resistance and the polarization resistance, as presented by the linear increase of potential for all samples. Compared to the 40IrO_2_/SnO_2_, the cell voltage of 40IrO_2_/Co*_x_*Sn_1−*x*_O_2_ shows a superior performance. The 40IrO_2_/Co_0.2_Sn_0.8_O_2_ exhibits optimal activity at 1A cm^−2^, followed by the order of 40IrO_2_/Co_0.3_Sn_0.7_O_2_, 40IrO_2_/Co_0.1_Sn_0.9_O_2_, 40IrO_2_/SnO_2_, in that order. However, the OER performance of the 40IrO_2_/Co*_x_*Sn_1−*x*_O_2_ catalysts is still lower than that of unsupported IrO_2_ due to the lower amount of Ir loading. The sequence in OER performance at 1 A cm^−2^ is IrO_2_ (1.713 V) < 40IrO_2_/Co_0.2_Sn_0.8_O_2_ (1.748 V) < 40IrO_2_/Co_0.3_Sn_0.7_O_2_ (1.770 V) < 40IrO_2_/Co_0.1_Sn_0.9_O_2_ (1.812 V) < 40IrO_2_/SnO_2_ (1.847 V). Notably, the OER performance decreases when the Co-doping level reaches to the point *x* = 0.3. This could be attributed to the increasing impurity scattering centres with the enhancement of Co contents that impede the electron transport and decrease the carrier mobility, leading to the degradation of OER performance [41].

**Figure 8.**
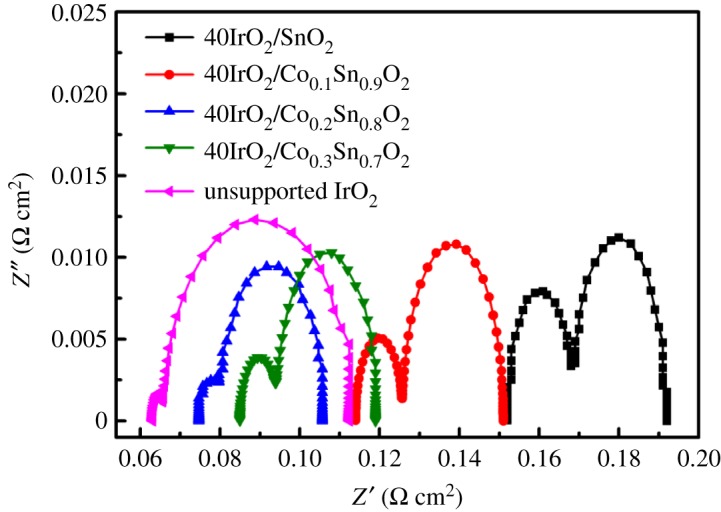
Nyquist diagrams of 40IrO_2_/Co*_x_*Sn_1−*x*_O_2_ (*x* = 0, 0.1, 0.2, 0.3) and unsupported IrO_2_ at 0.3 A cm^−2^ and 80°C.

The charge transfer resistance (R_ct_) is a critically important parameter in reflection of reaction kinetics in electrocatalytic performance for a catalyst, and a lower R_ct_ implies a faster reaction rate [43]. To further reveal the intrinsic charge transfer properties of IrO_2_ supported by varying Co-doped SnO_2_ as supports, the EIS measurements were conducted at 0.3 A cm^−2^ in the single cells. Figure 8 exhibits the Nyquist plots of 40IrO_2_/Co*_x_*Sn_1−*x*_O_2_ and unsupported IrO_2_, and the appropriate equivalent circuit model is shown in the inset. R_ct_ is the charge transfer resistance of a faradic process occurring at the interface of catalyst and electrolyte, which is evaluated from the large semicircles by the difference between low- and high-frequency intercepts on the real axis [44]. The ohmic resistance (*R*_Ω_) is the series resistance of all the geometry compositions in a cell. *R*_Ω_ can be calculated from the intercept extending from the high-frequency side of the curve of the real axis [45]. The calculated values of the *R*_ct_ and *R*_Ω_ are listed in table 2. It can be seen that the unsupported IrO_2_ shows a lower *R*_Ω_ (63.1 mΩ cm^2^) because of the excellent electrical conductivity than 40IrO_2_/Co*_x_*Sn_1−*x*_O_2_. The Rct value of 40IrO_2_/Co_0.2_Sn_0.8_O_2_ is 32 mΩ cm^2^ and exhibits the lowest charge transfer resistance, followed by 40IrO_2_/Co_0.3_Sn_0.7_O_2_ (34 mΩ cm^2^), 40IrO_2_/Co_0.1_Sn_0.9_O_2_ (37 mΩ cm^2^), 40IrO_2_/SnO_2_ (40 mΩ cm^2^) and unsupported IrO_2_ (52 mΩ cm^2^) in sequence. It is anticipated that the significant increase of the exposed surface area of IrO_2_ could enhance surface active sites and charge transport property, leading to the improvement of OER performance of 40IrO_2_/Co*_x_*Sn_1−*x*_O_2_.

**Figure 9.**
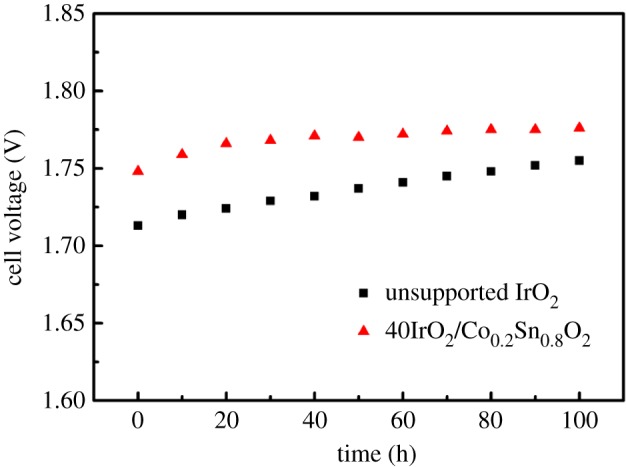
Durability test of the unsupported IrO_2_ and 40IrO_2_/Co_0.2_Sn_0.8_O_2_ sample in a single cell at 1 A cm^−2^ at 80°C.

Long-term durability is a critical parameter for the commercialization of catalysts. The durability test of the unsupported IrO_2_ and 40IrO_2_/Co_0.2_Sn_0.8_O_2_ catalysts in a single cell was measured at current densities of 1 A cm^−2^ at 80°C for 100 h. As displayed in figure 9, both the cell potentials of the samples reveal different degrees of increase. The cell potential of the unsupported IrO_2_ shows a relatively uniform upward trend, rising from 1.713 to 1.755 V after 100 h, and the average decay is 0.42 mV h^−1^. The cell voltage of 40IrO_2_/Co_0.2_Sn_0.8_O_2_ increases slightly during the initial 40 h, but later remains almost constant at 1.766 V, and the average degradation rate is 0.18 mV h^−1^. According to Kötz [46], the alternation between Ir(III) and Ir(IV) during the catalysis of OER plays a crucial role in the oxidation of the hydroxyl, while Ir(VI) is easily prone to corrosion according to the following [47]:

3.2 $$\mathrm{Ir}O_{2}+H_{2}O\to {\mathrm{IrO}}_{4}^{2-}+2H^{+}.$$

This implies that the supports are beneficial in the formation of stable Ir oxide during the modified Adams fusion, and that Co-doped content might further enhance the durability of 40IrO_2_/Co*_x_*Sn_(1−*x*)_O_2_ catalyst. Additionally, recent studies on OER performance and stability of iridium-based catalysts have been summarized and listed in table 3. It can be seen that the cell voltage and degradation rate of 40IrO_2_/Co_0.2_Sn_0.8_O_2_ are comparable to or superior to those of the previously reported iridium-based catalysts (such as IrO_2_/V-doped TiO_2_ [31] and IrO_2_-ATO [48]). This further confirms that the prepared Co-doped SnO_2_ as a support for IrO_2_ catalysts is a potential candidate for the practical application of SPE water electrolyzer.

**Table 3.** OER performance and stability reported in the literature for iridium-based catalysts.

references	anode catalyst	Ir loading (mg cm^−2^)	cathode catalyst	Pt loading (mg cm^−2^)	operating current (A cm^−2^)	operating temperature (°C)	cell voltage	electrode fabrication process	active area (cm^2^)	test time (h)	degradation rate (μV h^−1^)
Hao *et al*. [31]	IrO_2_/V -doped TiO_2_	2.5	Pt/C	0.5	1	80	2.015 V (1 A cm^−2^)	spraying	3.65	50	1980
Puthiyapura *et al*. [48]	IrO_2_-ATO	2	Pt/C	0.5	1.0	80	1.80 V (1 A cm^−2^)	spraying	1		
Rakousky *et al*. [49]	IrO_2_ and TiO_2_	2.25	Pt/C	0.8	2.0	80	1.84 V (2 A cm^−2^)	commercial	17.64	1150	194
Zeng *et al*. [50]	Ir black	0.873	Pt/C	1.0	0.25	80	1.728 V (2 A cm^−2^)	spraying	4	300	52
Faustini *et al*. [51]	Ir_0.7_Ru_0.3_O*_x_*	1.8	Pt/C	0.5	1	80	1.680 V (1 A cm^−2^)	spraying	6.25		
Jorge *et al*. [52]	gCNH-IrO2	1.2	Pt/C	4	1	80	1.93 V (1 A cm^−2^)	spraying	7.07		
this study	IrO_2_/Co_0.2_Sn_0.8_O_2_	2.5	Pt/C	0.5	1	80	1.776 V (1 A cm^−2^)	spraying	3.65	100	180

## 4. Conclusion

A varying amount of Co-doped SnO_2_ as anode support for IrO_2_ has been successfully prepared and then characterized by series methods. The particle sizes of the prepared Co*_x_*Sn_(1−*x*)_O_2_ (*x* = 0.1, 0.2, 0.3) samples were decreased with the increase of Co-doping content, which provided more sites for the well-dispersed IrO_2_. Also, the prepared samples exhibited increased high specific surface areas. The 40IrO_2_/Co_0.2_Sn_0.8_O_2_ exhibited the lowest overpotential with a cell potential of 1.748 V at 1000 mA cm^−2^ and showed a good stability during the 100 h operating at the current density 1 A cm^−2^ at 80°C. The decreased overpotential of 40IrO_2_/Co_0.2_Sn_0.8_O_2_ could be ascribed to the increment of surface active sites, the enhancement of electrical conductivity and the higher charge transfer, as verified by CVs and EIS measurement results. Consequently, the Co*_x_*Sn_(1−*x*)_O_2_ shows a promising alternative support for anode catalysts in the SPE water electrolyzer.

## Supplementary Material

EDS analysis of unsupported IrO2 and Cyclic voltammetry of CoxSn1-xO2

Reviewer comments

## Data accessibility

The datasets supporting this article have been uploaded as the electronic supplementary material.
