# Supplementary material for: Mesoporous CoxSn(1–x)O2 as an efficient oxygen evolution catalyst support for SPE water electrolyzer
Source: R Soc Open Sci. 2019 Apr 24;6(4):182223. doi: 10.1098/rsos.182223 (PMC6502374; doi:10.1098/rsos.182223)
Supplement: EDS analysis of unsupported IrO2 and Cyclic voltammetry of CoxSn1-xO2 [file rsos182223supp1.docx]

**Supporting Information**

Mesoporous Co_x_Sn_(1-x)_O_2_ as an efficient oxygen evolution catalyst support for SPE water electrolyzer

**Gang Chen,**^a^* Jiakun Li*^ac^* Hong Lv,**^bc^* Sen Wang,*^bc^* Jian Zuo,*^bc^* Lihua Zhu *^a^***

*a College of Materials and Engineering, Hunan University, Changsha, Hunan 410082, People's Republic of China. E-mail: chengang@hnu.edu.cn*

*b School of Automotive Studies, Tongji University, Shanghai 201804, People's Republic of China.* *E-mail: [lvhong@tongji.edu.cn](mailto:lvhong@tongji.edu.cn)*

*c Clean Energy Automotive Engineering Center, Tongji University, Shanghai 201804, People's Republic of China*


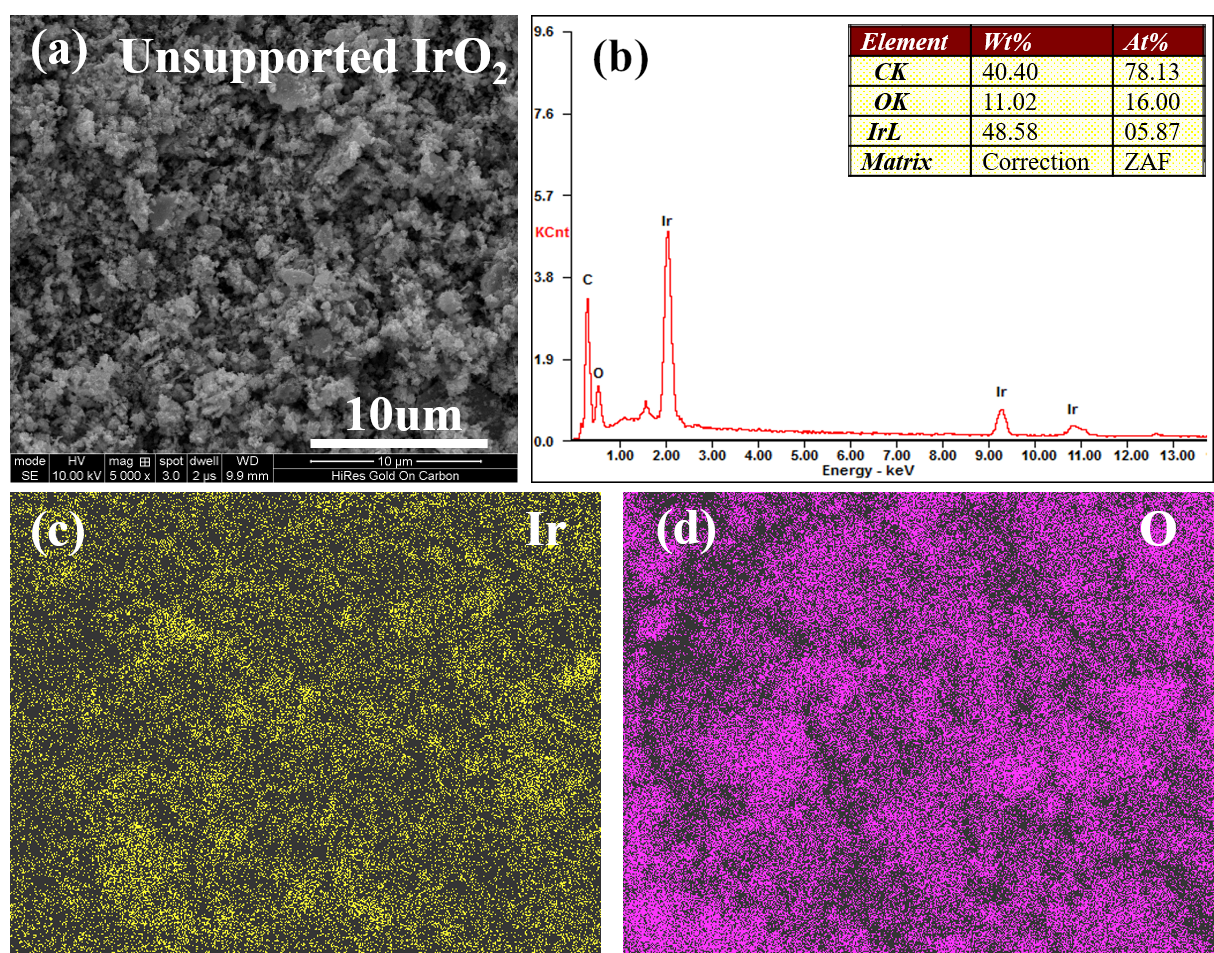


**Fig. S1** EDS analysis of unsupported IrO_2_; (a) SEM micrograph of the selected area for the mapping; (b) the resulting EDX spectrum; (c), (d) the elemental maps for Ir and O, respectively (the carbon signal corresponds to the sticky carbon tape used to trap the sample on the SEM samples holder).


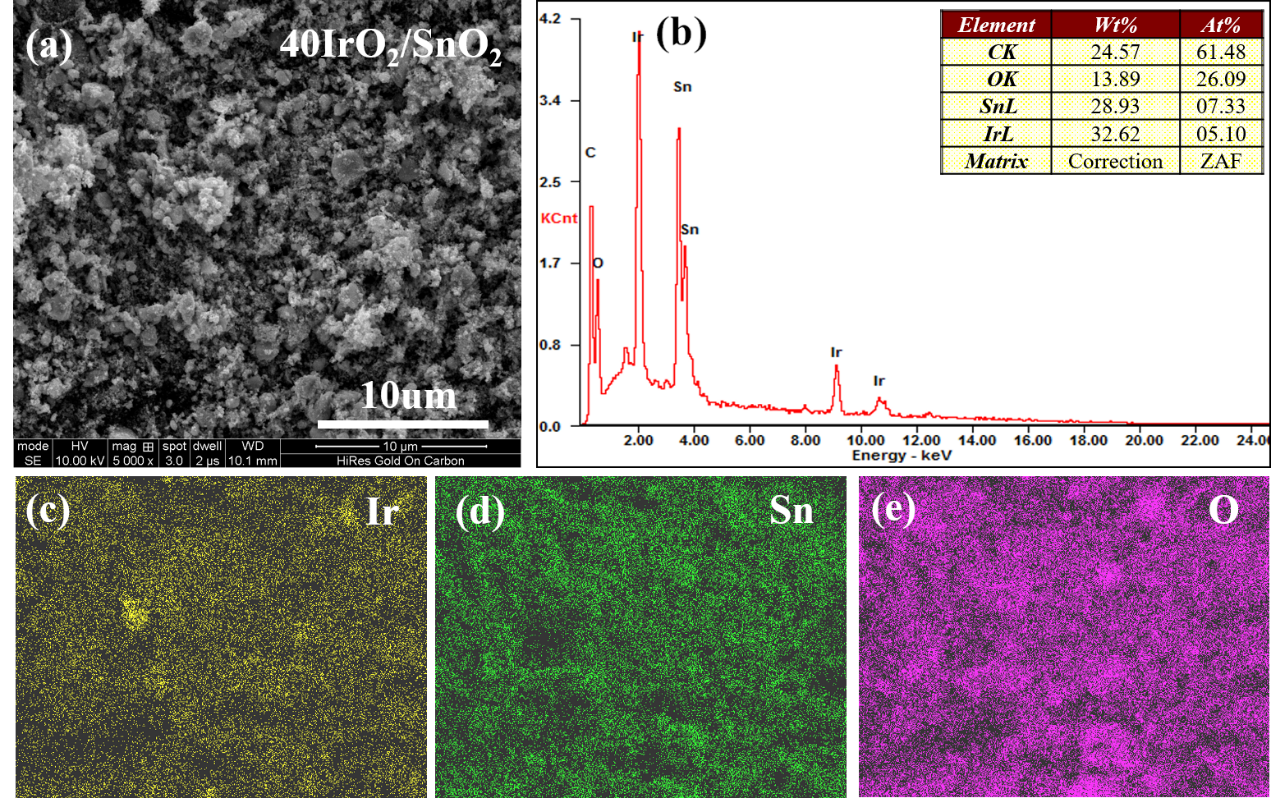


**Fig. S2** EDS analysis of 40IrO_2_/SnO_2_; (a) SEM micrograph of the selected area for the mapping; (b) the resulting EDX spectrum; (c)-(e) the elemental maps for Ir, Sn and O, respectively (the carbon signal corresponds to the sticky carbon tape used to trap the sample on the SEM samples holder).


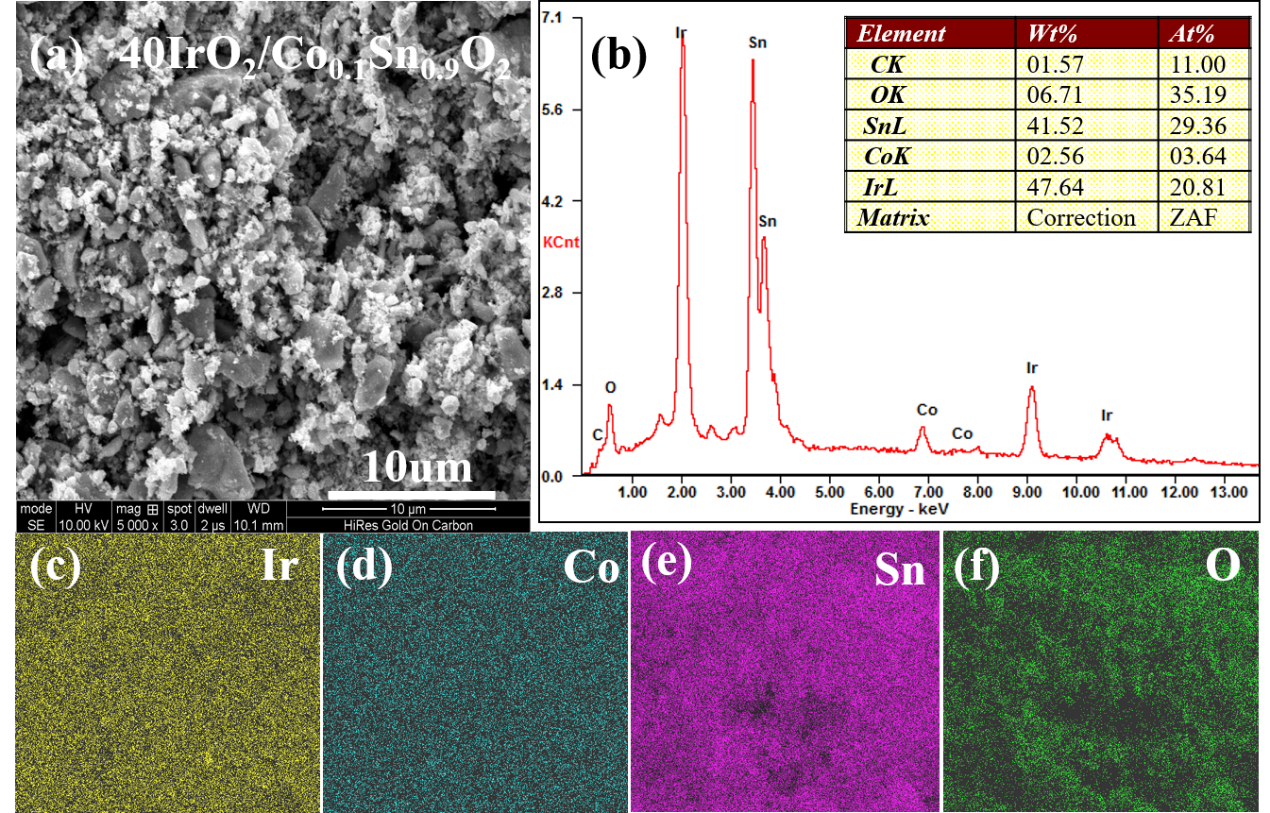


**Fig. S3** EDS analysis of 40IrO_2_/Co_0.1_Sn_0.9_O_2_; (a) SEM micrograph of the selected area for the mapping; (b) the resulting EDX spectrum; (c)-(f) the elemental maps for Ir, Co, Sn and O, respectively (the carbon signal corresponds to the sticky carbon tape used to trap the sample on the SEM samples holder).


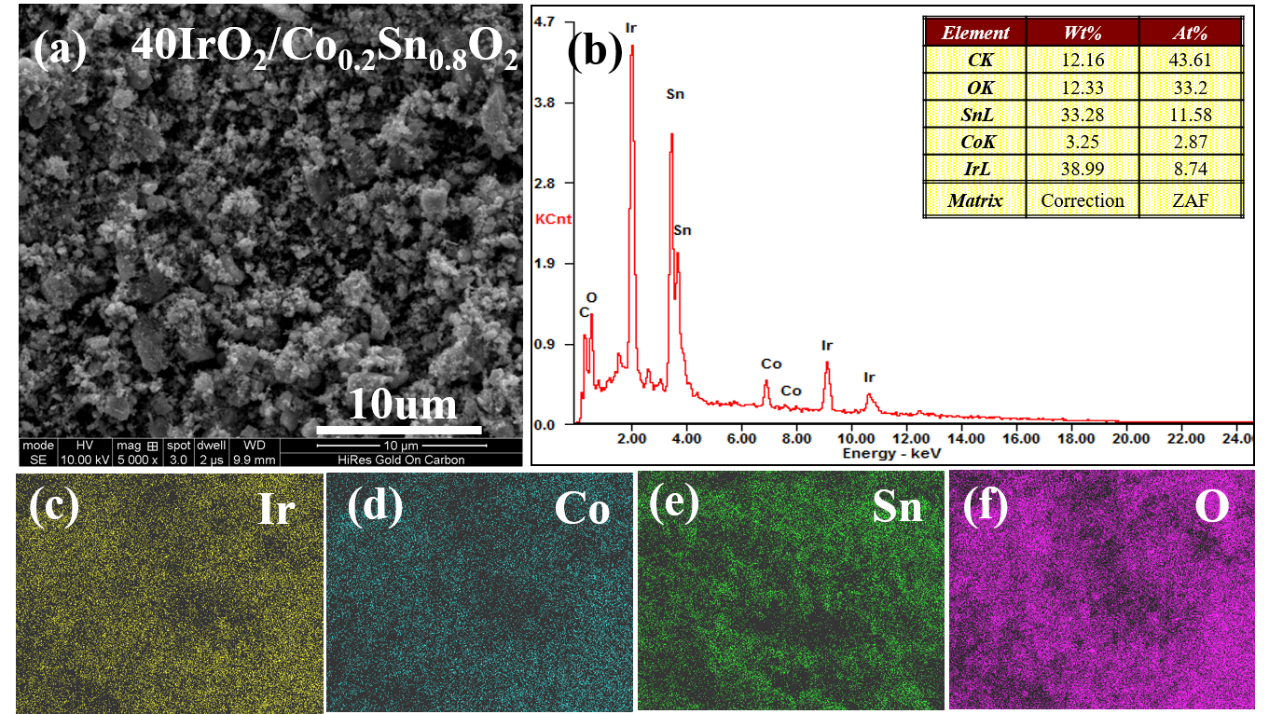


**Fig. S4** EDS analysis of 40IrO_2_/Co_0.2_Sn_0.8_O_2_; (a) SEM micrograph of the selected area for the mapping; (b) the resulting EDX spectrum; (c)-(f) the elemental maps for Ir, Co, Sn and O, respectively (the carbon signal corresponds to the sticky carbon tape used to trap the sample on the SEM samples holder).


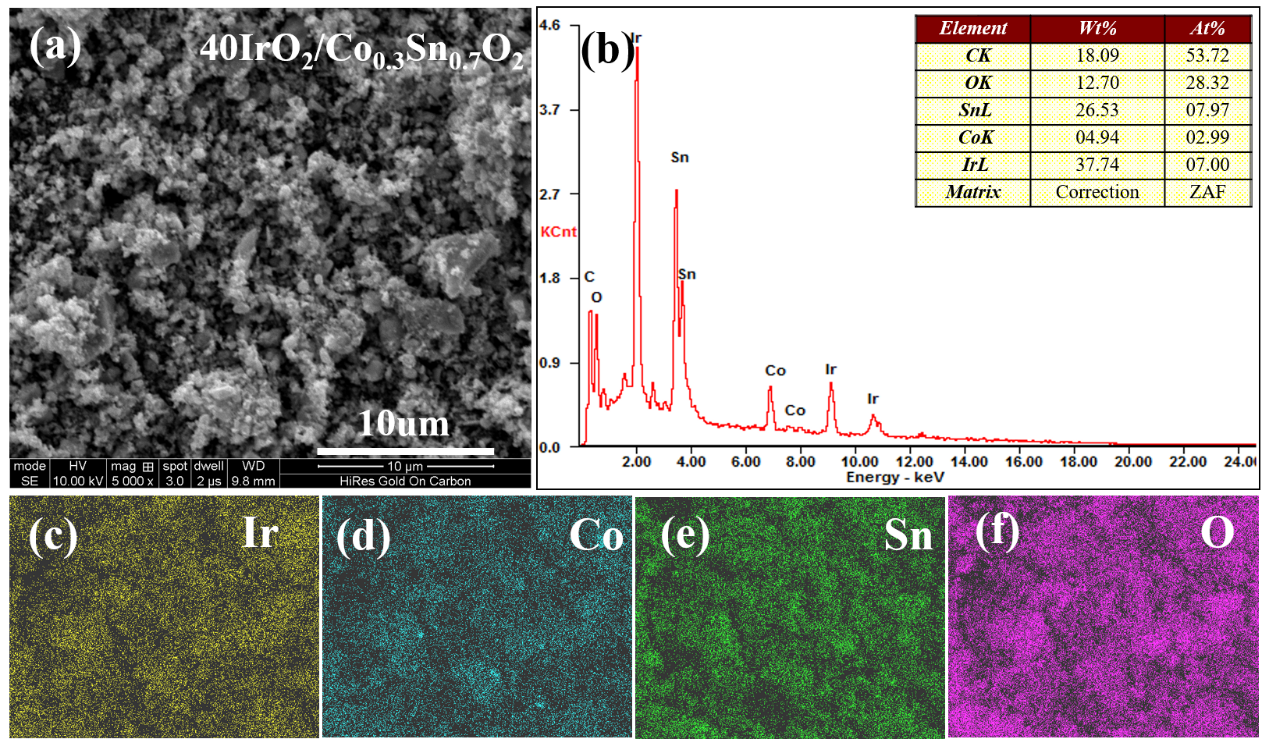


**Fig. S5** EDS analysis of 40IrO_2_/Co_0.3_Sn_0.7_O_2_; (a) SEM micrograph of the selected area for the mapping; (b) the resulting EDX spectrum; (c)-(f) the elemental maps for Ir, Co, Sn and O, respectively (the carbon signal corresponds to the sticky carbon tape used to trap the sample on the SEM samples holder).








**Fig. S6** (a) Cyclic voltammetry of Co_x_Sn_1-x_O_2_; (b) Linear sweep voltammetry of Co_x_Sn_1-x_O_2_





**Fig. S7** N_2_ adsorption isotherms and pore size distributions (inset) of the 40IrO_2_/Co_x_Sn_1-x_O_2_ samples.

**Table S1.** The BET surface area and BJH adsorption average results of the 40IrO_2_/Co_x_ Sn_(1-x)_ O_2_ (x= 0, 0.1, 0.2, 0.3).

| Samples | BET surface area (m^2^**·**g^-1^) | BJH adsorption average pore diameter (nm) |
| --- | --- | --- |
|  |  |  |
| 40IrO_2_/SnO_2_ | 53.21 | 4.74 |
| 40IrO_2_/Co_0.1_Sn_0.9_O_2_ | 87.54 | 6.86 |
| 40IrO_2_/Co_0.2_Sn_0.8_O_2_ | 91.25 | 7.65 |
| 40IrO_2_/Co_0.3_Sn_0.7_O_2_ | 93.06 | 6.47 |

**Table S2.** The electrical conductivity of supports Co_x_Sn_1-x_O_2_, catalysts 40IrO_2_/Co_x_Sn_1-x_O_2_ (x= 0, 0.1, 0.2, 0.3) and unsupported IrO_2_.

| Samples | Conductivity ( S cm^-1^) |
| --- | --- |
|  |  |
| SnO_2_ | 1.95×10^-6^ |
| Co_0.1_Sn_0.9_O_2_ | 2.02·10^-5^ |
| Co_0.2_Sn_0.8_O_2_ | 9.51·10^-5^ |
| Co_0.3_Sn_0.7_O_2_ | 6.94·10^-5^ |
| 40IrO_2_/SnO_2_ | 6.08·10^-2^ |
| 40IrO_2_/Co_0.1_Sn_0.9_O_2_ | 3.15·10^-1^ |
| 40IrO_2_/Co_0.2_Sn_0.8_O_2_ | 1.17·10^0^ |
| 40IrO_2_/Co_0.3_Sn_0.7_O_2_ | 8.13·10^-1^ |
| Unsupported IrO_2_ | 1.02·10^1^ |
